# Efficacy of Potassium-Competitive Acid Blockers to Treat Chronic Cough Associated With “Proven” Laryngopharyngeal Reflux Disease: A Preliminary Study

**DOI:** 10.7759/cureus.92757

**Published:** 2025-09-19

**Authors:** Takeshi Suzuki, Yosuke Seki, Tomoaki Matsumura, Jun Ikari, Tomoya Kurokawa, Mai Fujie, Syuji Yonekura, Toyoyuki Hanazawa, Toshitaka Hoppo

**Affiliations:** 1 Department of Otolaryngology, Head and Neck Surgery, Graduate School of Medicine, Chiba University, Chiba, JPN; 2 Minimally Invasive Surgery Center, Yotsuya Medical Cube, Tokyo, JPN; 3 Department of Gastroenterology, Graduate School of Medicine, Chiba University, Chiba, JPN; 4 Department of Respirology, Graduate School of Medicine, Chiba University, Chiba, JPN; 5 Clinical Research Center, Chiba University Hospital, Chiba, JPN; 6 Clinical Engineering Center, Chiba University Hospital, Chiba, JPN; 7 Division of General Surgery, Rutgers Robert Wood Johnson Medical School, New Brunswick, USA

**Keywords:** chronic cough, impedance tests, laryngopharyngeal reflux, laryngopharyngeal symptoms, potassium-competitive acid blocker

## Abstract

Introduction: Laryngopharyngeal reflux disease (LPRD) is thought to be a potential etiology of chronic cough (CC). A three-month empirical acid suppressive therapy has been recommended as a first-line diagnostic and therapeutic approach to treat patients with CC who are suspicious of LPRD, however its efficacy and benefit remain controversial. Since there has been no objective testing to accurately diagnose LPRD, patient cohorts in the previous studies are thought to be very heterogeneous. Therefore, the “true” efficacy of acid suppressive therapy for patients with CC and “proven” LPRD remains unknown. The objectives of this study were to assess the efficacy of potassium-competitive acid blocker (P-CAB) for “proven” LPRD-related CC and to see if P-CAB could be effective and beneficial in this setting.

Materials and methods: Patients with CC and LPRD as measured by hypopharyngeal-esophageal multichannel intraluminal impedance (HEMII), in whom empirical proton pump inhibitor (PPI) therapy previously failed, were enrolled, and daily P-CAB was given for 12 weeks. Cough Severity Index (CSI) and Reflux Symptom Index (RSI) scores were evaluated. Responders were defined as those who showed a 50.0% or more decrease in the CSI score.

Results: From February 2017 to December 2020, a total of 24 PPI-refractory patients with CC and LPRD were enrolled and analyzed. Of them, 15 patients (62.5%) had a normal acid exposure time. The CSI and RSI scores significantly improved from pre-P-CAB therapy (19.6 ± 9.8 and 18.5 ± 11.2, respectively) to post-P-CAB therapy (11.8 ± 11.1 and 14.1 ± 11.5, p = 0.003 and 0.024, respectively). Of 24 subjects, 14 (58.3%) subjects were responders, however, six (42.9%) subjects had abnormal RSI. Among responders to three-month P-CAB therapy, all subjects who discontinued P-CAB, and 50.0% of subjects who stayed on P-CAB, had recurrent cough within six months.

Conclusions: This preliminary study suggested that three-month P-CAB therapy appears to be a reasonable option for patients with CC and proven LPRD diagnosed by HEMII, even if PPI therapy fails and RSI is normal, however a long-term continuous P-CAB therapy may be required. More than half of patients had normal acid exposure, which may be missed by conventional pH-metry. Since a conventional pH-metry and RSI-based diagnosis of LPRD is insufficient, HEMII may be essential to evaluate patients with CC who are suspicious of LPRD.

## Introduction

Unexplained chronic cough (UCC) is defined as a cough that persists longer than eight weeks and remains unexplained even after appropriate investigation and treatment such as empirical acid suppressive therapy for gastroesophageal reflux disease (GERD) [1]. Because UCC causes significant impairments in quality of life, a practical algorithm of diagnosis and treatment is needed. It is essential to distinguish chronic cough (CC), which can be explained and effectively treated, from UCC [2], because incomplete investigation or inadequate treatment could also result in a misdiagnosis of UCC.

Laryngopharyngeal reflux (LPR), a retrograde flow of gastroduodenal contents up to the larynx and hypopharynx, can cause laryngopharyngeal symptoms (LPS) such as cough, voice hoarseness, throat clearing, excess phlegm in the throat, and sore throat. Currently, proton pump inhibitors (PPIs) and alginate in conjunction with diet and lifestyle modification are used as a first-line pharmacological therapy to treat patients with LPS, especially when a concomitant heartburn is present [3], however their “true” efficacy and benefit remain controversial. This is likely because there has been no objective testing to document the presence of LPR, and patients with LPS could therefore be too heterogeneous to draw any meaningful conclusions from previous clinical studies. Recently, the San Diego Consensus [3] stated that laryngopharyngeal reflux disease (LPRD) refers to patients with LPS and objective evidence of reflux, and the presence of LPS does not equate to LPRD. It is crucial to objectively document abnormal numbers of LPR events in patients with LPRD-related CC to accurately assess the “true” efficacy and benefit of any treatment options.

In general practice, the diagnosis of LPRD is often made based on symptom-based scores and/or laryngoscopic findings [4], however it has been well known that these diagnostic methods are not specific or reliable. We have utilized hypopharyngeal-esophageal multichannel intraluminal impedance (HEMII) to directly measure LPR events and document the presence of LPRD [5]. Based on the normative data established, the criteria of abnormal proximal exposure (APE) as measured by HEMII have been used as objective evidence of LPRD and integrated in the evaluation of patients with UCC. Previously, we have demonstrated that 73.1% of patients with UCC had APE, although half of the patients had negative acid exposure to the distal esophagus [6]. This suggested that a standard pH-metry may not be sufficient to exclude the possibility of reflux-related CC, and patients with UCC who are refractory to PPI therapy with diet and lifestyle modification might still include patients with LPRD-related CC, which could be treatable. Therefore, HEMII, which directly measures LPR events, is essential to evaluate patients with UCC.

A potassium-competitive acid blocker (P-CAB) has been introduced as a more effective, novel antisecretory medication for GERD than conventional PPIs. Therefore, P-CAB is currently considered to be the most effective and strongest acid-suppressive therapy for GERD in Japan [7]. The objectives of this preliminary study were to assess the outcome of P-CAB therapy in conjunction with diet and lifestyle modification in patients with CC and LPRD diagnosed by HEMII, and to see if P-CAB therapy could be effective and beneficial in this setting.

## Materials and methods

Study design

This preliminary study was conducted in accordance with the Declaration of Helsinki under the approval of the Institutional Review Board at the Chiba University School of Medicine (M10320,2356), and all participants gave written informed consent. Patient population in this study included those aged 18 or older with UCC diagnosed by pulmonologists and laryngologists, and had APE as measured by HEMII. All subjects had previously undergone at least two weeks of PPI therapy in conjunction with diet and lifestyle modification, without significant improvement in the clinical symptom of cough. For this study, all subjects were instructed to take P-CAB (vonoprazan fumarate 20mg) 30 minutes prior to breakfast for a maximum of 12 weeks. Dietary and lifestyle modifications were also recommended in conjunction with P-CAB. Exclusion criteria included those with medication noncompliance and with a known etiology of cough in the laryngopharyngeal area, such as laryngopharyngeal tumor, viral or bacterial infection in the airway or gastrointestinal tract, allergy, and postnasal drip. Medical therapy-resistant “adult-onset” asthma and interstitial pneumonia were not excluded because of their potential association with LPRD.

Detailed demographic data, subjective data (Cough Severity Index (CSI) and Reflux Symptom Index (RSI)), and objective data (endoscopic and HEMII findings) were obtained. The outcomes of P-CAB therapy were assessed using the CSI score. The prevalence of P-CAB responders was calculated, and adverse events were reviewed. Details of each objective testing and symptom questionnaires were described previously [8].

Objective testing

Upper endoscopy was performed by TM, a co-author of this study, as a primary experienced gastroenterologist. The severity of esophagitis was graded using the Los Angeles (LA) Classification [9].

**Figure 1:**
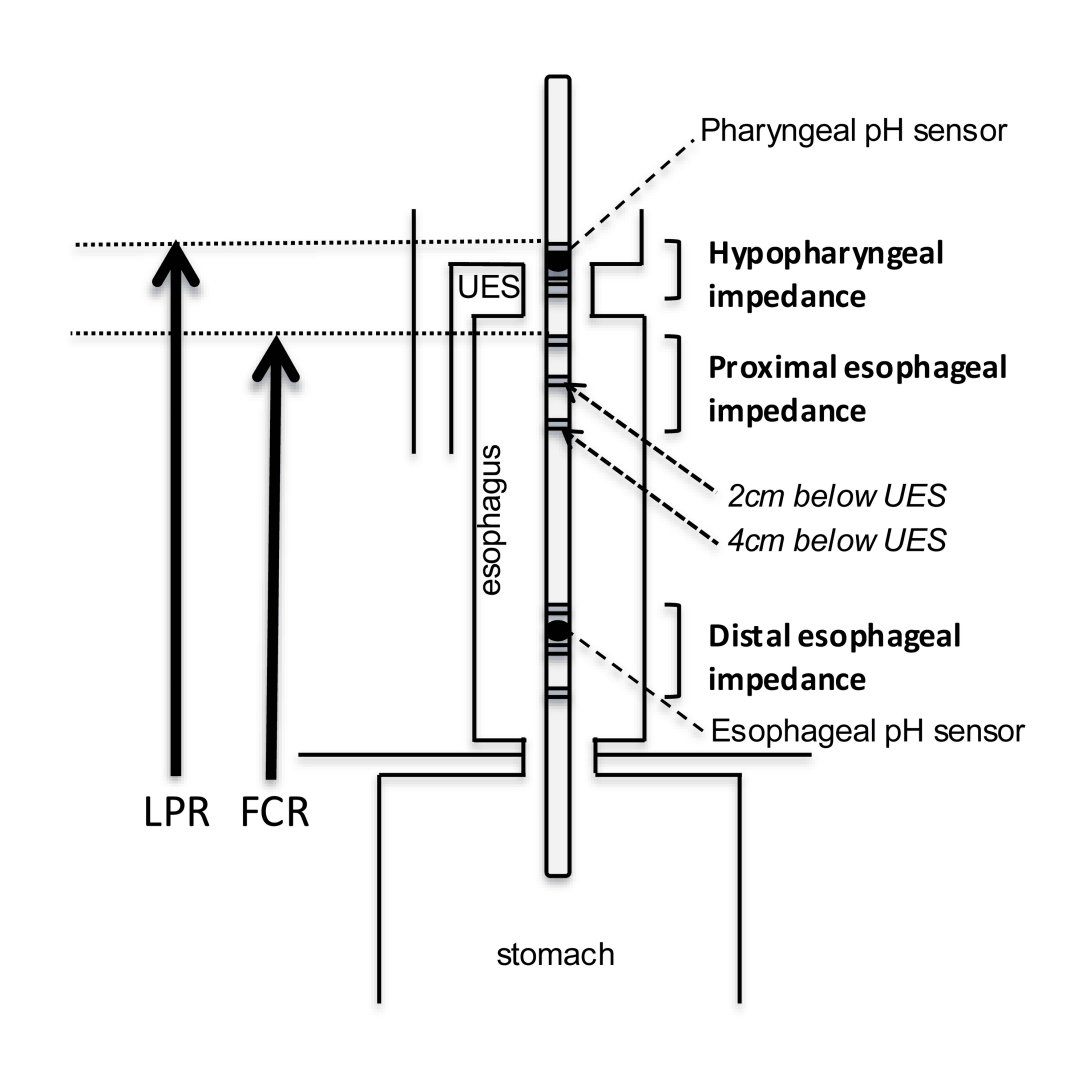
Hypopharyngeal-esophageal multichannel intraluminal impedance (HEMII) The catheter has two pairs of impedance electrodes in the distal esophagus, proximal esophagus and hypopharynx. LPR: laryngopharyngeal reflux; FCR: full column reflux; UES: upper esophageal sphincter Diagram created by the authors.

HEMII was performed using the specialized catheter (CAZI-BL-55,56; Diversatek, Highlands Ranch, CO, USA), which has two pairs of impedance electrodes in the distal esophagus, proximal esophagus and hypopharynx as shown in Figure 1 [10]. Antisecretory medications such as PPI and H2 antagonists were discontinued 10 days before HEMII. HEMII measurements were recorded for 24 hours. An LPR event was considered present when retrograde bolus transit occurred across all ring sets and reached the hypopharynx. Full column reflux (FCR) was defined as reflux that reached the impedance site 2 cm distal to the upper esophageal sphincter (UES) but did not reach the hypopharyngeal ring set. Based on the normative data established for LPR and FCR, APE was defined as LPR of one or more events per day and/or FCR of five or more events per day [11]. A diagnosis of LPRD was made when APE was positive on HEMII [6], providing objective evidence of reflux as stated in the San Diego Consensus [3].

Symptom questionnaire

To objectively assess and quantify the severity of cough and LPS, the validated objective questionnaires such as CSI (no definition of cutoff value) [12] and RSI (≥ 14 is abnormal) [13] (Appendix) were used. Generally, the patients with RSI ≥ 14 had “suggestive” LPRD. CSI is made up of 10 items which range from 0 (never) to 4 (always), with a maximum total score of 40, for patients with self-perceived symptoms of cough in order to quantify the severity of CC relating to upper airway disease. RSI is made up of nine items which range from 0 (no problem) to 5 (severe problem), with a maximum total score of 45. All subjects completed them the day before HEMII (off PPI), four, eight and 12 weeks after P-CAB therapy. Patients were divided into two groups: P-CAB responders and non-responders. Based on the criteria used in the previous study, responders were defined as those who showed a 50.0% or more decrease in CSI score [14]. All other patients were defined as non-responders. When a 90.0% or more decrease in the CSI score was observed, we defined that the cough disappeared.

Statistical analysis

Values are expressed as median (range or interquartile range) except for CSI and RSI scores as mean (SD). Statistical significance was determined by the Mann-Whitney test, the Wilcoxon signed-rank test and the Chi-square test using GraphPad Prism (version 9; GraphPad Software, La Jolla, CA, USA). Statistical tests were two-tailed, and a p-value < 0.05 was considered statistically significant.

## Results

Patient demographics

From February 2017 to December 2020, 24 patients with CC including refractory asthma (n = 6) and idiopathic interstitial pneumonia (n = 1), who were diagnosed with LPRD based on HEMII (11 men, 13 women), were enrolled in this study. None of the patients showed a significant symptomatic improvement with empirical PPI therapy and dietary and lifestyle modifications prior to this study. The median age and body mass index were 65.5 years (range, 25-82) and 22.5 (range, 18.6-38.7), respectively. Fifteen patients (62.5%) presented solely with cough, and nine (37.5%) patients had concomitant GERD symptoms such as heartburn and regurgitation. Fifteen (62.5%) patients had abnormal RSI scores. These demographics are shown in Table 1.

**Table 1:** Patient demographics * indicates p < 0.05, representing a statistically significant difference between responders and non-responders. Abbreviations: BMI, body mass index; GERD, gastroesophageal reflux disease; LA, Los Angeles; CSI, Cough Severity Index; RSI, Reflux Symptom Index

	No./total (%), median (range)			
	Total	Responders	Non-responders	
Characteristic	n = 24	n = 14	n = 10	P Value
Sex				
Male	11/24 (45.8%)	5/14 (35.7%)	6/10 (60.0%)	0.41
Female	13/24 (54.2%)	9/14 (64.3%)	4/10 (40.0%)	
Age, y	65.5 (25-82)	67.0 (42-82)	62.5 (25-79)	0.32
BMI	22.5 (18.6-38.7)	25.0 (20.7-30.5)	22.2 (18.6-38.7)	0.21
Concomitant GERD symptoms	9/24 (37.5%)	5/14 (35.7%)	4/10 (40.0%)	1.00
Duration of clinical symptoms, y	54.0 (3-400)	20.5 (3-156)	110 (10-400)	< 0.01 *
Esophageal mucosal injury				
LA grade				
N	20/22 (90.9%)	12/13 (92.3%)	8/9 (88.9%)	1.00
A	2/22 (9.1%)	1/13 (7.7%)	1/9 (11.1%)	
Hiatal hernia	7/22 (31.8%)	4/13 (30.8%)	3/9 (33.3%)	1.00
2 cm < Size ≤ 4 cm	2/7	2/4	-	-
Size ≤ 2 cm	5/7	2/4	3/3	
CSI, mean ± SD	19.6 ± 9.8	18.8 ± 9.8	20.8 ± 10.3	0.61
RSI, mean ± SD	18.5 ± 11.2	14.1 ± 10.9	24.6 ± 8.8	0.025 *
RSI ≥ 14	15/24 (62.5%)	6/14 (42.9%)	9/10 (90.0%)	0.03 *

Of 22 patients who had endoscopy, reflux esophagitis (LA-A) and hiatal hernia (< 4 cm) were found in two (9.1%) and seven (31.8%) patients, respectively. None of them had histologically confirmed Barrett’s esophagus. Table 2 presents HEMII data. Of 24 patients who had APE measured by HEMII, 23 patients (95.8%) and 11 patients (45.8%) had an abnormal number of FCR events (10 (2 - 33)) and LPR events (0 (0 - 6)), respectively. However, an abnormal number of total reflux events (≥ 80) was found only in six patients (25.0%). Fifteen patients (62.5%) had a normal acid exposure time (AET). A positive symptom-association probability (≥ 95.0%) was seen in five patients (20.8%).

**Table 2:** HEMII measurements Abbreviations: HEMII, hypopharyngeal-esophageal multichannel intraluminal impedance; FCR, full column reflux; LPR, laryngopharyngeal reflux

No./total (%), median (range)				
	Total	Responders	Non-responders	
HEMII Measurements	n = 24	n = 14	n = 10	P Value
No. of reflux events	54.5 (35-138)	53.0 (35-108)	60.0 (35-108)	0.85
No. of FCR events	10.0 (2-33)	12.5 (2-33)	8.5 (5-23)	0.14
No. of LPR events	0 (0-6)	1.0 (0-6)	0 (0-3)	0.19
Acid exposure time > 6.0%				
Positive	9/24 (37.5%)	4/14 (28.6%)	5/10 (50.0%)	0.39
Negative	15/24 (62.5%)	10/14 (71.4%)	5/10 (50.0%)	
Symptom association probability				
Positive	5/24 (20.8%)	3/14 (21.4%)	2/10 (20.0%)	1.00
Negative	19/24 (79.2%)	11/14 (78.6%)	8/10 (80.0%)	
Acid exposure time	4.9 (0.1-22.0)	3.4 (0.3-13.0)	6.9 (0.1-22.0)	0.51

Efficacy of P-CAB therapy

**Figure 2:**
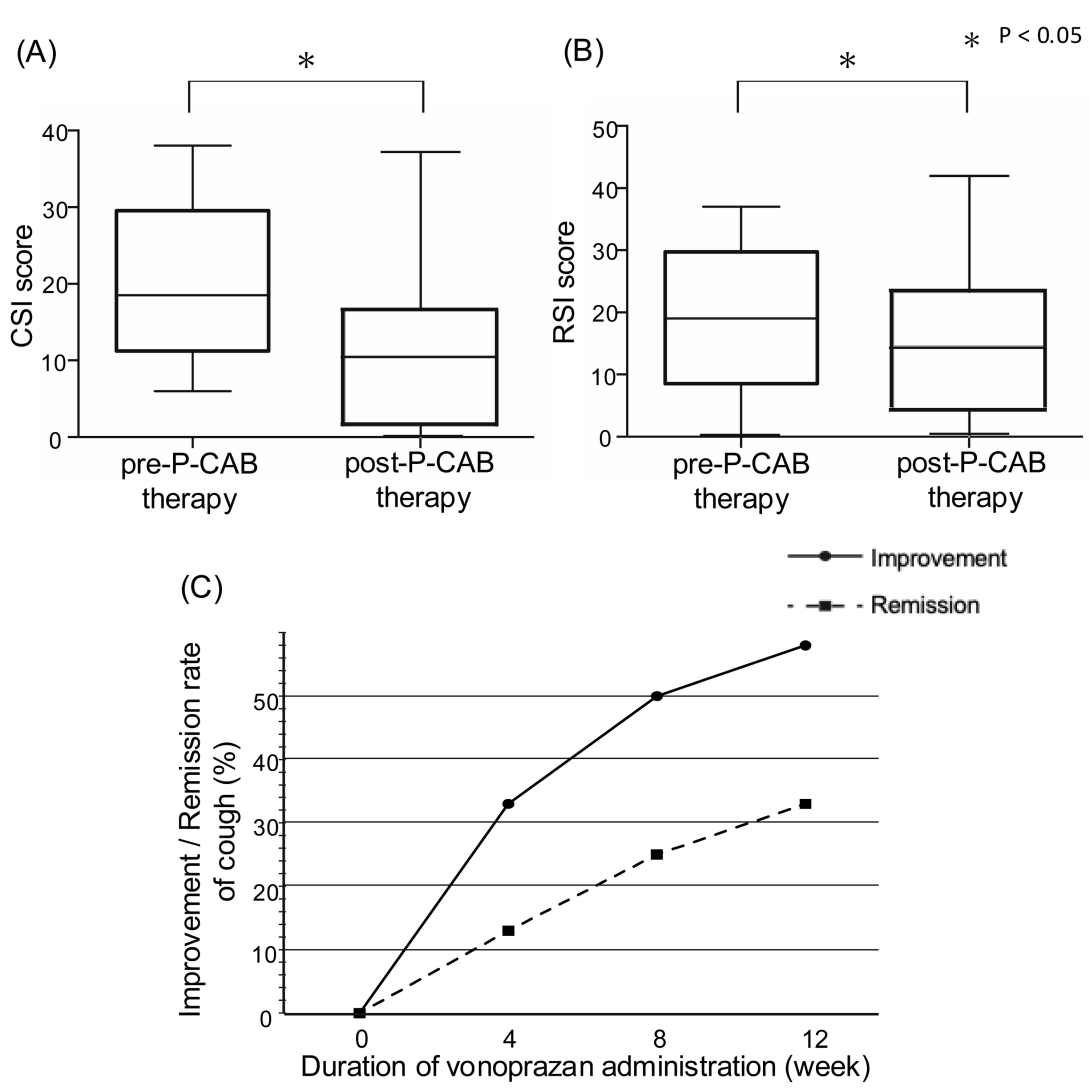
Efficacy of P-CAB therapy Comparison of the CSI (A) and RSI (B) score between pre- and post-P-CAB therapy. The CSI and RSI score were significantly lower at post-P-CAB therapy than pre-P-CAB therapy (CSI: 19.6 ± 9.8 vs. 11.8 ± 11.1, p = 0.003; RSI: 18.5 ± 11.2 vs. 14.1 ± 11.5, p = 0.024). Improvement and remission rate of cough during P-CAB therapy (C). * indicates p < 0.05, representing a statistically significant difference between pre- and post-treatment. CSI, Cough Severity Index; RSI, Reflux Symptom Index; P-CAB, potassium-competitive acid blocker

The CSI and RSI scores significantly improved from pre-P-CAB therapy (19.6 ± 9.8 and 18.5 ± 11.2, respectively) to post-P-CAB therapy (11.8 ± 11.1 and 14.1 ± 11.5, p = 0.003 and 0.024, respectively) at four weeks (14.4 ± 10.5 and 14.8 ± 10.6), eight weeks (13.9 ± 11.3 and 14.1 ± 12.3) and 12 weeks (11.7 ± 11.7 and 13.6 ± 11.6), as shown in Figure 2A, 2B. Of 24 patients with UCC and APE, 14 (58.3%) were considered responders to three-month P-CAB therapy. The number of P-CAB responders at four, eight and 12 weeks was eight (33.3%), 12 (50.0%) and 14 (58.3%). At pre-P-CAB therapy, only six (42.9%) out of 14 responders had abnormal RSI, whereas a majority of non-responders (90.0%) had abnormal RSI. Cough completely disappeared in three subjects (12.5%) at four weeks, six subjects (25.0%) at eight weeks, and eight subjects (33.3%) at 12 weeks, as shown in Figure 2C. One patient had mild constipation as an adverse event.

Comparison of patients with RSI score ≥ 14 and those with RSI score < 14

**Figure 3:**
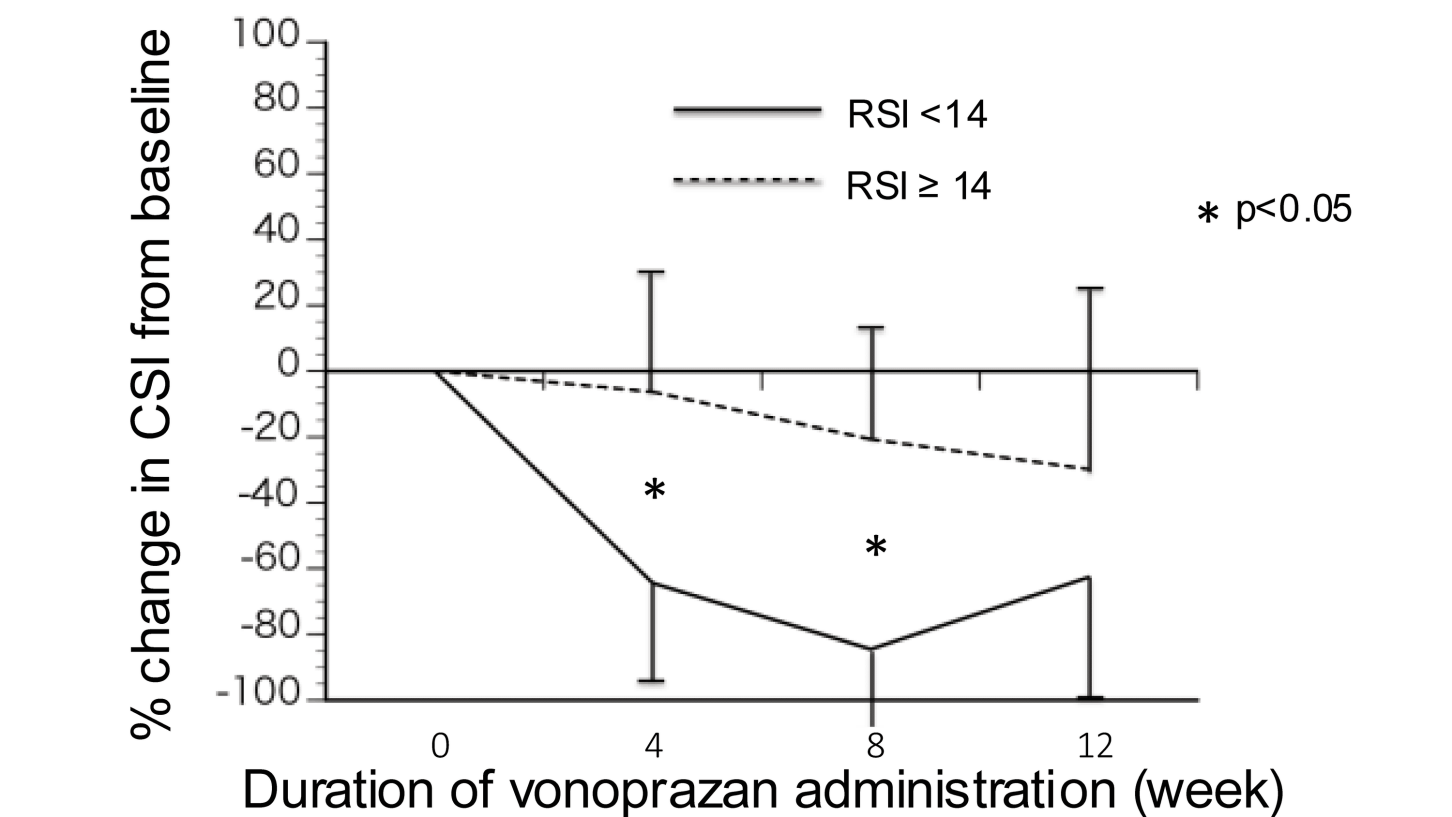
% change in CSI from baseline The % changes in CSI in the patients with RSI score ≥ 14 were significantly lower than those with RSI score < 14 at four weeks (-6.36 ± 36.7 vs -64.4 ± 32.2; p < 0.001) and eight weeks (-20.7 ± 35.0 vs -84.5 ± 23.0; p < 0.001), however significant difference was not observed at 12 weeks (-20.8 ± 55.8 vs -62.6 ± 35.9; p = 0.19). * indicates p < 0.05, representing a statistically significant difference between the patients with RSI score ≥ 14 and those with RSI score < 14. CSI, Cough Severity Index; RSI, Reflux Symptom Index

Figure 3 shows the % change in CSI score from baseline in the patients with RSI score ≥ 14 (n = 15) and with RSI score < 14 (n = 9), since RSI score ≥ 14 is generally suggestive of LPRD [13]. The % changes in CSI in the patients with RSI score ≥ 14 were significantly lower than those with RSI score < 14 at four weeks (-6.36 ± 36.7 vs -64.4 ± 32.2; p < 0.001) and eight weeks (-20.7 ± 35.0 vs -84.5 ± 23.0; p < 0.001), however no significant difference was found at 12 weeks (-20.8 ± 55.8 vs -62.6 ± 35.9; p = 0.19).

Outcomes of responders after three-month P-CAB therapy

Of 14 responders, six patients discontinued P-CAB, and all of them had recurrent cough within six months. Of six patients who decided to stay on the same initial dose of P-CAB, three patients had recurrent cough even with P-CAB during the six-month follow-up, whereas three patients did not have recurrent cough. One patient lost follow-up.

## Discussion

In this preliminary study, we demonstrated that 58.3% of PPI-refractory patients with CC and LPRD based on HEMII had significant symptomatic improvement after three-month P-CAB therapy. There appeared to be a tendency that those with RSI ≥14 were less responsive to P-CAB therapy compared to those with normal RSI. Furthermore, a majority of responders had recurrent cough not only after discontinuation of P-CAB but also even on continuous P-CAB therapy. Interestingly, 62.5% of patients with LPRD had a normal AET, which could be missed by conventional pH-metry. Based on the ERS guideline for UCC, these patients are therefore categorized as adult patients with UCC and a negative GERD workup, for whom PPI therapy is not recommended. Furthermore, in the absence of typical GERD symptoms, a standard 24-hour esophageal multichannel intraluminal impedance-pH monitoring (MII-pH) is considered to be of limited value [1]. This suggests that a conventional pH-metry, even a standard MII-pH, may not be sufficient to diagnose LPRD, and PPI-refractory patients with UCC and a negative GERD workup may still include those with LPRD-related CC. Therefore, HEMII may be an essential diagnostic tool to evaluate patients with UCC for optimal management.

In the present study, we used vonoprazan as P-CAB. The current PPIs are prodrugs that require acid activation and irreversibly inhibit gastric H+,K+-ATPase activity, the so-called proton pump. On the other hand, P-CAB does not need acid to inhibit gastric H+,K+-ATPase activity by reversible K+-competitive binding, which makes it possible to raise gastric pH more highly and effectively than PPIs [15]. The pH > 4 holding time of vonoprazan was higher than that of conventional PPIs (63.3% in vonoprazan 20mg vs. 39.1% in lansoprazole 30mg, 42.8% in rabeprazole 20mg and 43.1% in esomeprazole 40mg) [15]. Compared with conventional PPIs, vonoprazan provides a longer acid-inhibitory effect with an elimination half-life of up to nine hours, whereas that of PPIs is one to two hours [16]. Moreover, vonprazan accumulates higher and clears more slowly from gastric glands due to its higher pKa than other P-CABs [17]. Based on these data, vonoprazan could provide more profound and longer-lasting suppression of gastric acid secretion compared with either PPIs or other P-CABs [18] and has been used especially for PPI-resistant GERD and non-erosive gastroesophageal reflux disease [19,20]. Therefore, we used vonoprazan in the present study as the most effective, strongest acid-suppressive medication to date.

In the recent pilot study to compare the efficacy of esomeprazole and vonoprazan in patients with GERD-related CC, Zhong et al. reported that vonoprazan may be comparable to esomeprazole in relieving cough symptoms during a two-month treatment period, and suggested that this duration might be insufficient to fully assess the therapeutic effect on cough, especially considering the esophageal-bronchial reflex involved in GERD-related cough [21]. In this pilot study, a standard MII-pH was used, and patients with positive DeMeester scores, increased number of reflux episodes and/or positive symptoms association probability were included for analysis. However, not all patients had positive DeMeester scores, which has been used as conclusive data of GERD, while an increased number of reflux episodes or positive symptoms association probability are supportive, but inconclusive data for GERD [22]. Furthermore, even patients who had only symptom-based diagnoses without objective testing were included. This suggested the patient cohort in this pilot study was still very heterogeneous, and this could affect the results of the analysis. In contrast, the patient cohort in our preliminary study consisted of highly selected patients with CC and LPRD as documented by HEMII, in all of whom empirical PPI therapy had previously failed. Therefore, the strength of the present study was that a “true” efficacy of P-CAB was assessed on highly selected patients with CC and “proven” LPRD. Since 58.3% of patients were responders to P-CAB, three-month P-CAB therapy could be a reasonable option to treat LPRD-related CC even if PPI therapy fails. However, a large number of patients had recurrent cough after discontinuation of P-CAB or even with continuous P-CAB therapy. Similar to PPI, P-CAB changes the acidity of gastric contents but does not stop reflux events [23]. Moreover, potential adverse effects of P-CAB such as hypomagnesemia, hypergastrinemia with enterochromaffin-like cell hyperplasia, and renal injury have been suggested, although long-term safety data on P-CAB remain limited [7,16,24]. It is therefore thought that antireflux surgery (ARS) may be more reasonable option in this setting, to prevent any potential adverse effects of long-term P-CAB use. This was further supported by the recent retrospective review of patients with proven LPRD diagnosed by HEMII, which demonstrated that ARS using magnetic sphincter augmentation achieved favorable improvement in LPS in 80.4% of cases, with cough symptoms also showing significant improvement [25].

Since the larynx is a sensitive organ, chronic LPR, regardless of its acidity, could continuously irritate the laryngopharyngeal epithelium, eventually developing its hypersensitivity [26]. Once laryngopharyngeal hypersensitivity is acquired, even subtle stimulants other than LPR could induce atypical laryngopharyngeal symptoms [8]. In the present study, cough recurred in 50.0% of responders even on continuous P-CAB therapy. Also, those with RSI ≥ 14 appeared to be less responsive to P-CAB compared to those with normal RSI. These findings could be explained by laryngopharyngeal hypersensitivity. Yiming et al. [27] demonstrated that, among the patients with GERD-related CC diagnosed by MII-pH, the efficacy of a routine PPI dose plus prokinetic agents in the RSI < 19 group was markedly better than that in the RSI ≥ 19 group (P = 0.009). Moreover, additional use of neuromodulators such as gabapentin or baclofen in the RSI ≥ 19 group achieved better efficacy than the RSI < 19 group (P = 0.026 in baclofen + PPI, P = 0.010 in gabapentin + PPI). This suggested that PPI therapy may be insufficient for patients with GERD-related CC and preexisting hypersensitivity, and that the addition of a neuromodulator could be more efficient. On the other hand, increased sensitivity of P2X3 receptors, which are expressed on airway vagal afferent nerves, could mediate sensitization of the cough reflex and could be a potential cause of refractory cough. Therefore, P2X3 antagonist has been introduced as a promising treatment for CC induced by neuronal hypersensitivity [1]. Most importantly, it is crucial to appropriately and timely evaluate patients with CC before laryngopharyngeal hypersensitivity is developed as it could be refractory to any type of treatment.

In this preliminary study, the sample size was small. We used RSI instead of Reflux Symptom Score (RSS), which is currently proven to be more accurate, because this trial was initiated before the superiority and accuracy of RSS over RSI was reported. Also, the efficacy of vonoprazan was not compared with conventional PPIs, although empirical PPI therapy failed prior to P-CAB therapy in all subjects. A larger-scale study with long-term follow-up data is required to confirm our preliminary findings.

## Conclusions

This preliminary study suggested that three-month P-CAB therapy would be a reasonable option for patients with CC and proven LPRD diagnosed by HEMII, even if PPI therapy fails, however a long-term continuous P-CAB therapy may be required. Since PPI dependence, conventional pH-metry and RSI may not be sufficient to diagnose LPRD, HEMII could be an essential testing for optimal management of patients with UCC. It is important to appropriately and timely evaluate patients with CC before laryngopharyngeal hypersensitivity is developed, as it could be refractory to any type of treatment.

## Appendices

**Table 3:** Cough Severity Index (CSI) CSI is made up of 10 items, each ranging from 0 (never) to 4 (always), with a maximum total score of 40. [12]

Item	Question	0	1	2	3	4
1	I have a persistent cough	□	□	□	□	□
2	My cough interrupts my conversation	□	□	□	□	□
3	My cough affects my ability to talk on the telephone	□	□	□	□	□
4	My cough affects my ability to participate in social activities	□	□	□	□	□
5	My cough is worse when I am lying down	□	□	□	□	□
6	My cough wakes me up at night	□	□	□	□	□
7	My cough causes me to feel anxious or stressed	□	□	□	□	□
8	My cough causes me to avoid public places	□	□	□	□	□
9	My cough causes physical discomfort or pain	□	□	□	□	□
10	My cough causes me to feel tired or fatigued	□	□	□	□	□

**Table 4:** Reflux Symptom Index (RSI) RSI is made up of nine items, each ranging from 0 (no problem) to 5 (severe problem), with a maximum total score of 45. [13]

Item	Question	0	1	2	3	4	5
1	Hoarseness or a problem with your voice	□	□	□	□	□	□
2	Clearing your throat	□	□	□	□	□	□
3	Excess throat mucus or postnasal drip	□	□	□	□	□	□
4	Difficulty swallowing food, liquids, or pills	□	□	□	□	□	□
5	Coughing after you ate or after lying down	□	□	□	□	□	□
6	Breathing difficulties or choking episodes	□	□	□	□	□	□
7	Troublesome or annoying cough	□	□	□	□	□	□
8	Sensation of something sticking in your throat or a lump in your throat	□	□	□	□	□	□
9	Heartburn, chest pain, indigestion, or stomach acid coming up	□	□	□	□	□	□
